# Pou2f2 Regulates the Distribution of Dorsal Interneurons in the Mouse Developing Spinal Cord

**DOI:** 10.3389/fnmol.2019.00263

**Published:** 2019-11-07

**Authors:** Gauhar Masgutova, Audrey Harris, Benvenuto Jacob, Lynn M. Corcoran, Frédéric Clotman

**Affiliations:** ^1^Université catholique de Louvain, Institute of Neuroscience, Laboratory of Neural Differentiation, Brussels, Belgium; ^2^Université catholique de Louvain, Institute of Neuroscience, System and Cognition Division, Brussels, Belgium; ^3^Molecular Immunology Division and Immunology Division, The Walter and Eliza Hall Institute, Parkville, VIC, Australia

**Keywords:** embryonic spinal cord, dorsal interneurons, *Pou2f2*, onecut, neuronal migration

## Abstract

Spinal dorsal interneurons, which are generated during embryonic development, relay and process sensory inputs from the periphery to the central nervous system. Proper integration of these cells into neuronal circuitry depends on their correct positioning within the spinal parenchyma. Molecular cues that control neuronal migration have been extensively characterized but the genetic programs that regulate their production remain poorly investigated. Onecut (OC) transcription factors have been shown to control the migration of the dorsal interneurons (dINs) during spinal cord development. Here, we report that the OC factors moderate the expression of *Pou2f2*, a transcription factor essential for B-cell differentiation, in spinal dINs. Overexpression or inactivation of *Pou2f2* leads to alterations in the differentiation of dI2, dI3 and Phox2a-positive dI5 populations and to defects in the distribution of dI2-dI6 interneurons. Thus, an OC-Pou2f2 genetic cascade regulates adequate diversification and distribution of dINs during embryonic development.

## Introduction

The dorsal spinal cord relays and processes somatosensory inputs, nociception, thermosensation, pruriception, mechanosensation and proprioception, from peripheral sensory neurons to central targets. Furthermore, somatosensory perception is crucial for fine regulation and proper execution of motor activities. The complex organization of these neural circuits requires precise spatial position of neuronal cell bodies and integration into proper connectivity routes (Lu et al., 2015; Lai et al., 2016). Neuronal positioning and the cellular and molecular mechanisms involved in this process have been extensively studied in the developing brain (Marín et al., 2010). Although the molecules directing neuronal migration in the developing spinal cord start to be identified (Andrews et al., 2006; Kim et al., 2015; Junge et al., 2016; Leggere et al., 2016), much less is known about the genetic programs that control this essential process.

During embryonic development, eight populations of dorsal interneurons (dINs) are produced from progenitor domains orderly distributed along the dorso-ventral (DV) axis of the ventricular zone in the dorsal spinal cord. A unique combinatorial code of basic helix-loop-helix (bHLH) or homeodomain transcription factors defines, in two neurogenic waves, six early dIN populations (dI1–dI6) generated between embryonic day (e) 10.5 and e12.5, and two late-born dIL^A^ and dIL^B^ INs produced between e11 and e13 (Caspary and Anderson, 2003; Helms and Johnson, 2003). When specified, dINs migrate long distance along the DV axis to their distinctive location and integrate into specific circuits (Hernandez-Miranda et al., 2017). Among the early-born dINs, the dI1 excitatory INs localize in the intermediate spinal cord (Bermingham et al., 2001) while some dI2 excitatory INs migrate towards the intermediate layers of the spinal cord and others invade the ventral horn (Gross et al., 2002). The intermediate and deep dorsal horn contains glutamatergic dI3 (Bui et al., 2013). The inhibitory dI4 INs settle laterally in the deep dorsal horn (Gross et al., 2002; Müller et al., 2002; Pillai et al., 2007) and excitatory dI5 INs invade the deep dorsal and the ventral horns (Ding et al., 2004). Located in the ventromedial region of the spinal cord, dI6 INs give rise to Dmrt3- or WT1-containing subsets (Lanuza et al., 2004; Andersson et al., 2012; Schnerwitzki et al., 2018). The two late-born dIL^A^ and dIL^B^ populations occupy the superficial laminae of the dorsal horn (Gross et al., 2000; Müller et al., 2002). During spinal cord development, these heterogeneous dorsal populations continue to diversify into small and discrete subsets (Ding et al., 2004; Del Barrio et al., 2013; Rosenberg et al., 2018) that each follows a stereotyped pattern of migration to reach their final location within the spinal parenchyma.

Spinal neuron distribution along the DV and mediolateral (ML) axes constitute a critical feature of microcircuit organization and functionality (Ladle et al., 2007; Tripodi et al., 2011). Indeed, proper cell body position of dorsal inhibitory INs along the ML axis is crucial for the establishment of their sensorimotor connectivity (Hilde et al., 2016) while the distribution of distinct Lbx1-positive premotor (Goetz et al., 2015) or V1 IN subsets (Bikoff et al., 2016) constrains patterns of input from sensory and motor neurons. In addition, V3 INs segregate into dorsal or ventral sub-populations that differ in their connectivity patterns and are active during distinct motor activities (Borowska et al., 2013, 2015; Chopek et al., 2018). Furthermore, segmental distinctions exist along the rostro-caudal axis of the spinal cord. Columnar organization of motor neurons varies between brachial or lumbar and thoracic regions in register with the part of the body these cells target (Francius and Clotman, 2014; Catela et al., 2015). In parallel, as several INs populations are associated with differential motor output, IN integration into local microcircuits is also influenced by their distribution along the antero-posterior axis (Bikoff et al., 2016; Hayashi et al., 2018; Sweeney et al., 2018). Even if the genetic networks orchestrating the production and the differentiation of spinal INs have been extensively deciphered (Lu et al., 2015), less is known about the transcription factors that dictate their distribution in the developing spinal cord and might consequently influence their connectivity profiles and spinal circuit formation. The Onecut (OC) transcription factors namely Hepatocyte Nuclear Factor-6 (HNF-6, or OC-1), OC-2 and OC-3, are detected in the digestive tract and in the CNS during embryonic development (Lemaigre et al., 1996; Landry et al., 1997; Jacquemin et al., 1999, 2003; Vanhorenbeeck et al., 2002). Besides their roles in the production (Espana and Clotman, 2012b), diversification (Roy et al., 2012; Francius and Clotman, 2014; Kabayiza et al., 2017) or maintenance (Espana and Clotman, 2012a, b; Stam et al., 2012) of specific neuronal populations, they also regulate neuronal distribution of various neuron types. OC regulate proper organization and migration of dopaminergic neurons of the A13 nucleus during their second phase of development (Espana and Clotman, 2012b). Similarly, they contribute to the reorganization of the Purkinje cells during a late phase of cerebellar development (Audouard et al., 2013). More recently, their contribution in the regulation of neuronal distribution was also uncovered in dorsal and in ventral spinal INs (Kabayiza et al., 2017; Harris et al., 2019). However, little is known about the downstream molecular effectors of the OC factors in dIN development, particularly regarding their migration along the DV and the ML axes of the spinal cord. In the present study, we uncovered that the expression of *Pou2f2*, a POU family transcription factor (Clerc et al., 1988; Corcoran et al., 1993), is controlled by OC factors in the spinal dINs. We show that Pou2f2 is present in dI2-dI6 populations during the early stages of development. Analysis of OC mutant embryos demonstrated that OC proteins moderate the expression of *Pou2f2* in the dorsal spinal cord. Using gain-of-function experiments, we observed that increased Pou2f2 modulates the distribution of the dI2-dI6 INs. Loss-of-function experiments confirmed that Pou2f2 regulates the localization of the dI2-dI6 and the number of cells in some dIN populations. Thus, Pou2f2 controls the differentiation and the distribution of dINs in the developing spinal cord.

## Materials and Methods

### Ethic Statement and Mouse Lines

All experiments were strictly performed in accordance with the European Community Council directive of 24 November 1986 (86–609/ECC) and the decree of 20 October 1987 (87-848/EEC). Mice were raised in our animal facilities and treated according to the principles of laboratory animal care. Experimental procedures and mouse housing were both approved by the Animal Welfare Committee of Université Catholique de Louvain (Permit Numbers: 2013/UCL/MD/11 and 2017/UCL/MD/008). Mutant strain mice were crossed and the day of vaginal plug was considered to be embryonic day (e) 0.5. The embryos were collected at e10.5, e11.5, e12.5 and e14.5. A minimum of three embryos of the same genotype was analyzed in each experiment. The *Hnf6;Oc2* and the *Pou2f2* mutant mice have been described previously (Corcoran et al., 1993; Jacquemin et al., 2000; Clotman et al., 2005). The *Hnf6;Oc2* mutant embryos additionally lack OC-3 in the developing spinal cord (Roy et al., 2012; Kabayiza et al., 2017).

### *In situ* Hybridization and Immunofluorescence Labelings

For *in situ* hybridization, embryos were fixed in ice-cold 4% paraformaldehyde (PFA) in phosphate buffered-saline (PBS) overnight at 4°C, washed thrice in PBS for 10 min, incubated in PBS/30% sucrose solution overnight at 4°C, embedded and frozen in PBS/15% sucrose/7.5% gelatin. Fourteen micrometer section were prepared and *in situ* hybridization was performed as previously described (Beguin et al., 2013; Pelosi et al., 2014; Francius et al., 2016) with DIG-conjugated Pou2f2 (NM_011138.1, nucleotides 604–1,187) antisense RNA probes. Control or *Hnf6/Oc2*^−/−^ mutant sections were placed adjacent on the same histology slides to minimize inter-slide variations of *in situ* hybridization signals. For immunofluorescence labelings, embryos were fixed in ice-cold 4% PFA in PBS for 10–35 min according to their embryonic stage, incubated in PBS/30% sucrose solution overnight at 4°C, embedded and frozen in PBS/15% sucrose/7.5% gelatin. Immunostaining was performed on 12 or 14 μm serial cryosections as previously described (Francius and Clotman, 2010). Primary antibodies against the following proteins were used: Brn3a (mouse 1:1,000; Santa Cruz #sc-8429), Dmrt3 (guinea pig; 1:1,000; kindly provided by K. Kullander #170), Foxd3 (guinea pig; 1:1,000; or rabbit; 1:1,000; kindly provided by T. Müller), Foxp1 (goat; 1:1,000; R&D Systems #AF4534), HNF6 [guinea pig; 1:2,000; (Espana and Clotman, 2012a) or rabbit; 1:100; Santa Cruz #sc-13050 or sheep; 1:1,000 R&D Systems #AF6277], Isl1/2 (goat; 1:3,000; Neuromics #GT15051 or mouse; 1:6,000; DSHB #39.4D5), Lbx1 (guinea pig; 1:10,000 or rabbit; 1:5,000; kindly provided by T. Müller), Lhx1/5 (mouse; 1:1,000; DSHB #4F2), Lmx1b (guinea pig; 1:10,000 or rabbit; 1:2,000; kindly provided by T. Müller), OC2 [rat; 1:400; (Clotman et al., 2005) or sheep; 1:500; R&D Systems #AF6294], OC3 [guinea pig; 1:6,000; (Pierreux et al., 2004), Phox2a (rabbit; 1:500; kindly provided by J.-F. Brunet), Pou2f2 (rabbit; 1:2,000; Abcam #ab178679), Wt1 (rabbit; 1:2,000; Santa Cruz #sc-192)]. Secondary antibodies were the donkey anti-guinea pig/AlexaFluor 488, 594 or 647, anti-mouse/AlexaFluor 488, 594 or 647, anti-rabbit/AlexaFluor 594 or 647, anti-goat/AlexaFluor 488, anti-rat/AlexaFluor 488 or 647, anti-sheep/AlexaFluor 594, and goat anti-mouse IgG1 specific/AlexaFluor 488 or 594, anti-mouse IgG2A specific/AlexiaFluor 488, anti-mouse IgG2B specific/AlexaFluor 647, purchased from Thermo Fisher Scientific or Jackson Laboratories, and were used at dilution 1:1,000.

### *In ovo* Electroporation

*In ovo* electroporations were performed at stage HH14–16, as previously described (Roy et al., 2012). The coding sequence of *Pou2f2* was amplified by overlapping-PCR, as previously described *Harris* using: forward 5′ GCTCTGTCTGCCCAAGAGAAA 3′ and reverse 5′ GTTGGGACAAGGTGAGCTGT 3′ primers for the 5′ region, forward 5′ CCACCATCACAGCCTACCAG 3′ and reverse 5′ ATTATCTCGAGCCAGCCTCCTTACCCTCTCT 3′ (designed to enable integration at the *Xho*I restriction site of the pCMV-MCS vector) primers for the 3′ region. This sequence was first subcloned in a pCR^®^II-Topo^®^ vector (Life Technologies, 45–0640) for sequencing then subcloned at the *Eco*RI (from the pCR^®^II-Topo^®^ vector) and *Xho*I restriction sites of a pCMV-MCS vector for the *in ovo* electroporation. The pCMV-Pou2f2 (0.5 μg/μl) vector was co-electroporated with a pCMV-eGFP plasmid (0.25 μg/μl) to visualize electroporated cells. The embryos were collected 72 h (HH27-28) after electroporation, fixed in PBS/4%PFA for 45 min and processed for immunofluorescence labelings as previously described (Francius and Clotman, 2010).

### *In situ* Hybridization Signal Intensity Measurements

*In situ* hybridization images of cryosections were acquired on an EVOS FL Auto Cell Imaging System (Thermo Fisher Scientific, Waltham, MA, USA). For each embryo (*n* *=* 3), one side of five sections at lumbar level was quantified using ImageJ by delineating an area of 13,500 px^2^ using the “rectangular” selection tool and evaluating signal intensity using Measure under Analyze. Intensity of the background signal from an adjacent area devoid of labeling was subtracted to normalize for background variations. All numbers are arbitrary units. Raw data were exported from ImageJ software to Sigma Plot v12.3 software to perform statistical analysis. The histograms were drawn with Microsoft Excel. Adequate statistical test was applied based on the number of comparisons and on the variance in each group. For analysis of signal intensity based on comparison of two groups (control or *Hnf6/Oc2*^−/−^), a standard Student’s *t*-test was performed. Difference was considered as significant at *p* ≤ 0.05.

### Cell Quantification

Immnofluorescence images of cryosections were acquired on a Confocal Laser Scanning microscope FV1000 Fluoview with the FV10-ASW 01.02 software (Olympus) or an EVOS FL Auto Cell Imaging System (Thermo Fisher Scientific, Waltham, MA, USA). For each labeling, acquisition parameters were identical for control or mutant sections. Brightness and contrast were adjusted uniformly in all replicate panels within an experiment with Adobe Photoshop CS6 software to match with the observation. Quantifications were performed on red or green or blue layer of acquired confocal images and double or triple labeled cells were processed by subtractive method (Francius and Clotman, 2010). For each embryo (*n* *=* 3), one side of five sections at brachial, at thoracic and at lumbar levels were quantified using the count analysis tool of Adobe Photoshop CS6 software. Raw data were exported from Adobe Photoshop CS6 software to Sigma Plot v12.3 software to perform statistical analyses, and the histograms were drawn with Microsoft Excel. Adequate statistical tests were applied based on the number of comparisons and on the variance in each group. For analysis of cell quantifications based on comparison of two groups (control or mutant; control or electroporated side of the spinal cord), standard Student’s *t*-tests or Mann–Whitney *U* tests were performed. Differences were considered as significant at *p* ≤ 0.05.

### Spatial Distribution

Pictures for quantitative analyses of dorsal interneuron distribution were acquired on a Confocal Laser Scanning microscope FV1000 Fluoview with the FV10-ASW 01.02 software (Olympus). Spinal cord height (H) was defined as the distance from the ventral-most to the dorsal-most point of spinal cord, and width (W) as the distance from central canal to the most lateral edge (adapted from Palmesino et al., 2010). For each dIN, distance (D_IN_) and angle (α_IN_) were measured from the ventral-most point of the spinal cord to the interneuron soma using the ruler analysis tool in Adobe Photoshop CS6 software. DV and ML position of dINs were expressed as percentage of spinal cord height and hemicord width, respectively: DV position and ML position were defined as (D_IN_ * sinα_IN_)/H and (D_IN_ * cosα_IN_)/W, respectively (Palmesino et al., 2010). ML vs. DV values were plotted using Matlab software R2013a (Mathworks, Canada). Statistical analyses of dIN distribution were performed using a two-sample Hotelling’s T2, which is a two-dimensional generalization of the Student’s *t*-test, as described for similar data sets (Palmesino et al., 2010; Kabayiza et al., 2017; Harris et al., 2019). The analysis was implemented using the NCSS software package.

## Results

### OC Factors Moderate *Pou2f2* Expression in dIN Populations

The OC transcription factors control the distribution of dIN populations (Kabayiza et al., 2017). In an effort to identify genes downstream of OC involved in this process, we analyzed the results of a transcriptomic comparison between embryonic day (e)11.5 wildtype or OC-deficient spinal cords (GEO repository accession number: GSE117871; Harris et al., 2019). Surprisingly, we could not identify significant changes in expression of genes coding for demonstrated or potential migration cues, except for Draxin. However, expression pattern of *Draxin* or distribution of the corresponding protein was not changed in *OC* mutant spinal cords (data not shown). In contrast, we confirmed that the expression level of *Pou2f2*, a transcription factor containing a POU-specific domain and a POU-type homeodomain, was significantly increased in the spinal cord in the absence of OC proteins. *Pou2f2* is expressed in B-lymphocytes, in neuronal cell lines and in neural tissues (Hatzopoulos et al., 1990; Lillycrop and Latchman, 1992; Camós et al., 2014). It is required for differentiation of B lymphocytes and is able to modulate neuronal differentiation of ES cells (Corcoran et al., 1993, 2004; König et al., 1995; Theodorou et al., 2009; Hodson et al., 2016). The expression level of *Pou2f2* was 2.6-fold upregulated in *Hnf6/Oc2*^−/−^ spinal cords (Harris et al., 2019). However, a potential contribution of Pou2f2 to dIN development was unknown.

**Figure 1:**
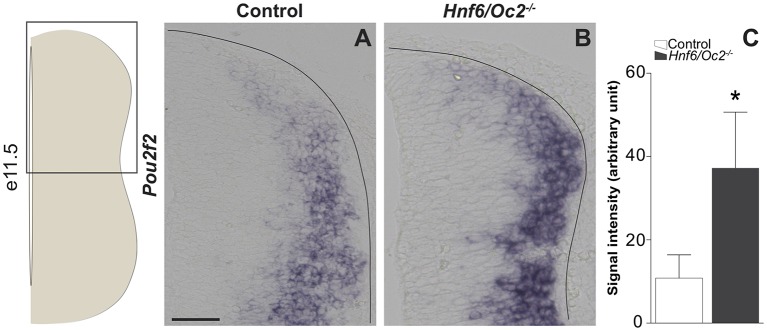
Onecut (OC) factors moderate expression of *Pou2f2* in the dorsal spinal cord.** (A,B)** *In situ* hybridization for *Pou2f2* on transverse sections (lumbar level) of control or *Hnf6/Oc2*^−/−^ spinal cords at e11.5. **(A)** In control embryos, *Pou2f2* is expressed at low levels in the dorsal part of the spinal cord and at higher levels in the intermediate zone. **(B)** In *Hnf6/Oc2*^−/−^ mutant embryos, *Pou2f2* expression is upregulated and cells displaying high *Pou2f2* levels are observed more dorsally than in control littermates. The pictures show part of right hemisections as indicated on the scheme to the left. **(C)** Measurement of *Pou2f2 in situ* hybridization signal intensity in control or *Hnf6/Oc2*^−/−^ at e11.5. *Pou2f2* expression is upregulated in the absence of the OC factors in the dorsal spinal cord (*p* ≤ 0.05). Mean values ± SEM, *n* = 3. * = *p* ≤ 0.05. Solid lines delineate the spinal cord. Scale bar = 100 μm.

Using *in situ* hybridization, we first confirmed that *Pou2f2* expression was increased in the dorsal part of the spinal cord in* Hnf6/Oc2*^−/−^ embryos. Consistent with quantifications on the whole spinal cord (Harris et al., 2019), we measured a ~3-fold increase in signal intensity in the absence of OC proteins (Figure 1). To assess the distribution of Pou2f2 in the dIN populations and to determine if the increase in *Pou2f2* expression was due to an expansion of Pou2f2 distribution or to an upregulation in its endogenous expression domain, we quantified the number of Pou2f2-containing dINs between e10.5 and e12.5 in wildtype and in OC-deficient embryos. As previously demonstrated, the OC factors are neither present in dI1 nor in dIL^A^ and dIL^B^ INs (Kabayiza et al., 2017). Moreover, due to the lack of antibodies for dI4 and early dI6 specific markers compatible with the Pou2f2 antibody species (see “Materials and Methods” section), we were unable to study the presence of Pou2f2 in those populations.

**Figure 2:**
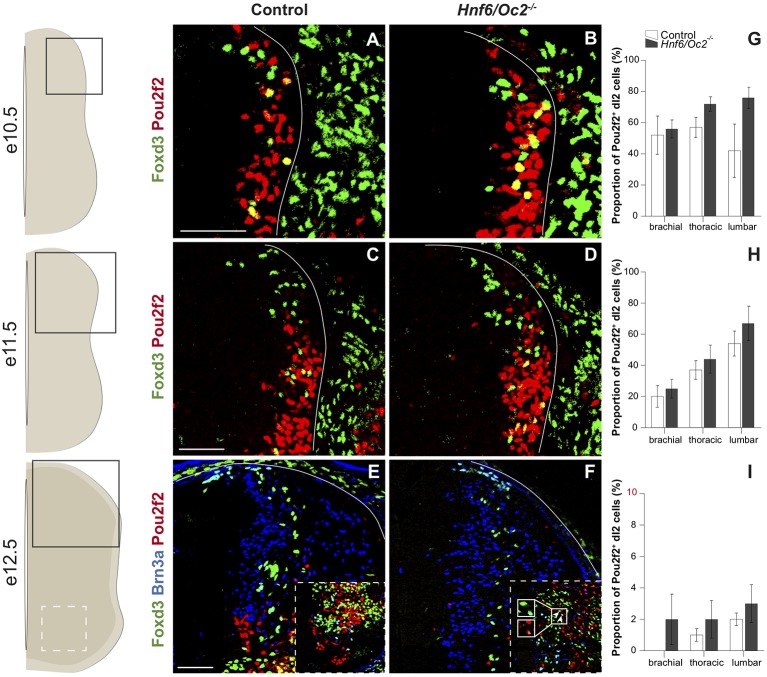
The OC factors moderate *Pou2f2* expression in dI2 interneurons.** (A–F)** Immunodetection of Pou2f2 (red) in Foxd3^+^ (green) or Foxd3^+^ (green) Brn3a^+^ (blue) dI2 interneurons on transverse sections of thoracic spinal cord at e10.5 **(A,B)**, e11.5 **(C,D)** and e12.5 **(E,F)**. Pou2f2 is detected in post-mitotic Foxd3^+^ dI2 interneurons, particularly in the ventral part of the population, at e10.5 and e11.5 but is almost absent at e12.5. Pou2f2 signal is stronger in Foxd3^+^ dI2 interneurons in *Hnf6/Oc2*^−/−^ spinal cord at all studied developmental stages. **(G–I)** Relative quantification of Pou2f2 positive dI2 neurons at e10.5 **(G)**, e11.5 **(H)** and e12.5 **(I)**. The proportion of Pou2f2^+^ dI2 is not significantly different in *Hnf6/Oc2*^−/−^ spinal cords. The pictures show part of right hemisections as indicated on the schemes to the left. Solid lines delineate the spinal cord. Insets in **(E,F)** are magnified views of boxed ventral regions. Arrowheads in **(F)** point to triple-labeled cells. Mean values ± SEM, *n* = 3. Scale bars = 100 μm.

Immunofluorescence analyses demonstrated that Pou2f2 is produced in post-mitotic dI2 INs, defined by the presence of Foxd3 at e10.5 and e11.5 and of Foxd3 and Brn3a at e12.5. In control embryos, Pou2f2 was detected in a significant fraction of a ventral dI2 cell contingent at e10.5 (Figures 2A,B) but, as observed for OC factors (Kabayiza et al., 2017), the proportion of dI2 Pou2f2-positive INs decreased at e11.5 (Figures 2C,D) and Pou2f2 was almost completely absent from dI2 cells at e12.5 (Figures 2E,F). In mutant embryos, the number of Pou2f2-positive dI2 trended to increase as compared to control embryos, but this change was not statistically significant. Labeling intensity was stronger in dI2 of *OC* mutant embryos (Figures 2A–F), consistent with the increased *Pou2f2* expression in the dorsal regions of the spinal cord.

**Figure 3:**
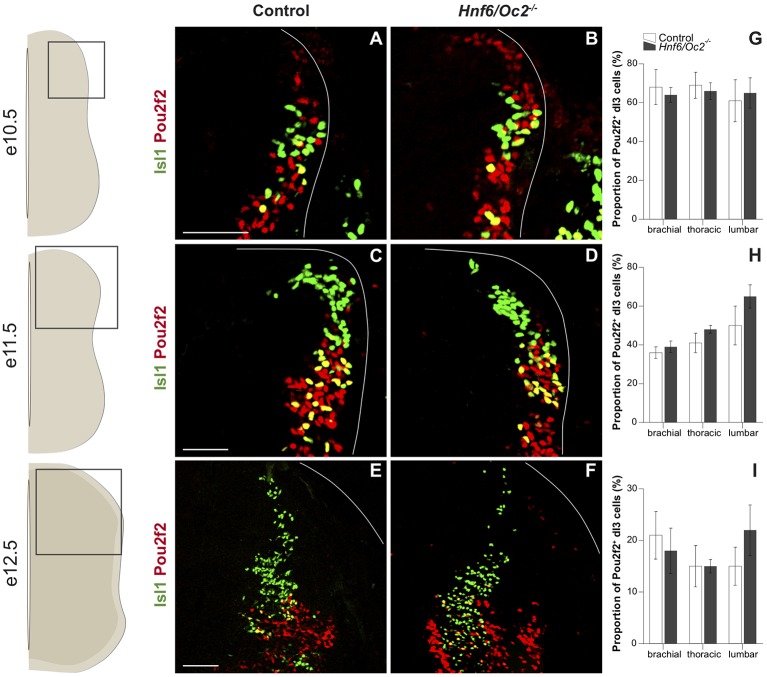
The OC factors moderate *Pou2f2* expression in dI3 interneurons.** (A–F)** Immunodetection of Pou2f2 (red) in Isl1^+^ (green) dI3 interneurons on transverse sections of thoracic spinal cord at e10.5 **(A,B)**, at e11.5 **(C,D)** and e12.5 **(E,F)**. Pou2f2 is detected in the ventral part of the post-mitotic Isl1^+^ dI3 interneuron population from e10.5. Pou2f2 signal is stronger in Isl1^+^ dI3 interneurons in *Hnf6/Oc2*^−/−^ spinal cord at all studied developmental stages. **(G–I)** Relative quantification of Pou2f2 positive dI3 neurons at e10.5 **(G)**, e11.5 **(H)** and e12.5 **(I)**. The proportion of Pou2f2^+^ dI3 is not significantly different in *Hnf6/Oc2*^−/−^ spinal cords. The pictures show part of right hemisections as indicated on the schemes to the left. Solid lines delineate the spinal cord. Mean values ± SEM, *n* = 3. Scale bars = 100 μm.

In dI3 INs, characterized by the presence of Isl1, Pou2f2 was detected in the ventral part of the population from the early stages of their development (Figures 3A,B). As observed for dI2 INs, the proportion of Pou2f2-positive dI3 cells progressively decreased as development proceeds (Figures 3C–I). Pou2f2 was detected in a similar proportion of dI3 cells in control and in mutant embryos between e10.5 and e12.5 (Figures 3G–I). Nonetheless, the signal intensity was stronger in *OC* mutant dI3 cells (Figures 3A–F).

**Figure 4:**
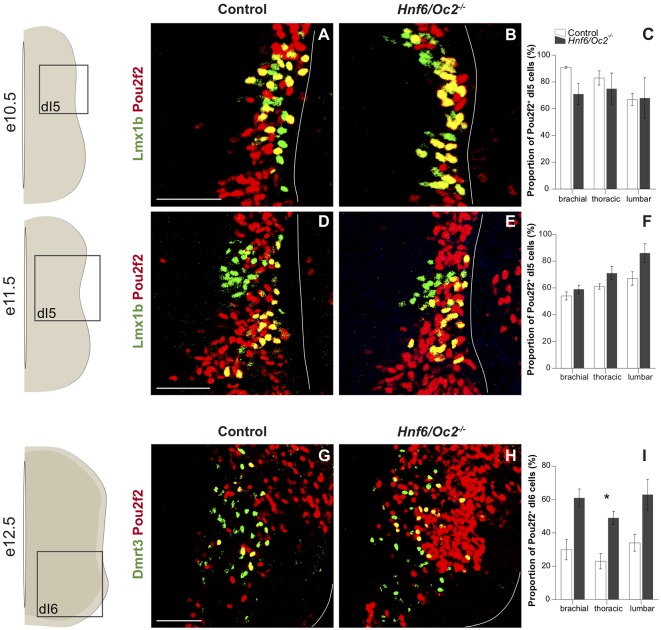
The OC factors moderate *Pou2f2* expression in dI5 interneurons and Dmrt3^+^ dI6 interneuron subset.** (A–D)** Immunodetection of Pou2f2 (red) in Lmx1b^+^ (green) dI5 neurons on transverse sections of thoracic spinal cord at e10.5 **(A,B)** and at e11.5 **(C,D)**. Pou2f2 is detected in most of Lmx1b^+^ dI5 interneurons at e10.5 and is then restricted to the ventral part of the population. Pou2f2 signal is stronger in Lmx1b^+^ dI5 interneurons in *Hnf6/Oc2*^−/−^ spinal cord at all studied developmental stages. **(E,F)** The percentage of Pou2f2 positive dI5 neurons was quantified at e10.5 **(E)** and e11.5 **(F)**, and is not significantly different in *Hnf6/Oc2*^−/−^ spinal cords. **(G–I)** Immunodetection and relative quantification of Pou2f2 in Dmrt3^+^ (green) dI6 subset at e12.5. The proportion of dI6 Dmrt3^+^ Pou2f2^+^ is significantly increased at thoracic level in *Hnf6/Oc2*^−/−^ mutant embryos (*p* ≤ 0.05). Again, Pou2f2 signal is stronger in the absence of OC factors. The pictures show part of right hemisections as indicated on the schemes to the left. Solid lines delineate the spinal cord. Mean values ± SEM, *n* = 3. * = *p* ≤ 0.05. Scale bars = 100 μm.

Lmx1b early-born dI5 INs were analyzed at e10.5 and e11.5 to distinguish them from late-born dIL^B^ also identified by Lmx1b at later stages. In control embryos, as for the other dIN populations, Pou2f2-positive cells represented a higher proportion of dI5 INs at e10.5, with most of the dI5 containing Pou2f2 (Figure 4A), while this proportion decreased at e11.5 (Figure 4C). In *Hnf6/Oc2*^−/−^ mutant embryos, the proportion of Pou2f2-positive dI5 cells trended to decrease at e10.5 (Figures 4A–C) and to increase at e11.5 (Figures 4D–F). However, these changes were not statistically significant (Figures 4C,F). Again, stronger intensity of the labeling at e11.5 confirmed increased Pou2f2 production in dI5 INs.

The lack of a unique specific marker for dI6 INs complicates the analysis of Pou2f2 in this population at early stages. Nevertheless, we were able to characterize the presence of Pou2f2 in the dI6 Dmrt3^+^ subset at e12.5 (Figures 4G–I). In control embryos, between 20% and 40% of dI6 cells contained Pou2f2. The proportion of dI6 Dmrt3^+^ producing Pou2f2 trended to increase in mutant compared to control embryos, although the change was statistically significant only at thoracic level (Figure 4I). Moreover, as for the other dorsal populations, Pou2f2 signal was stronger in *Hnf6/Oc2*^−/−^ mutant embryos. Taken together, these observations suggest that OC factors temper Pou2f2 expression in dINs and restrain its expression to a subset of dI6 INs.

### *Pou2f2* Overexpression Alters the Distribution of dINs

**Figure 5:**
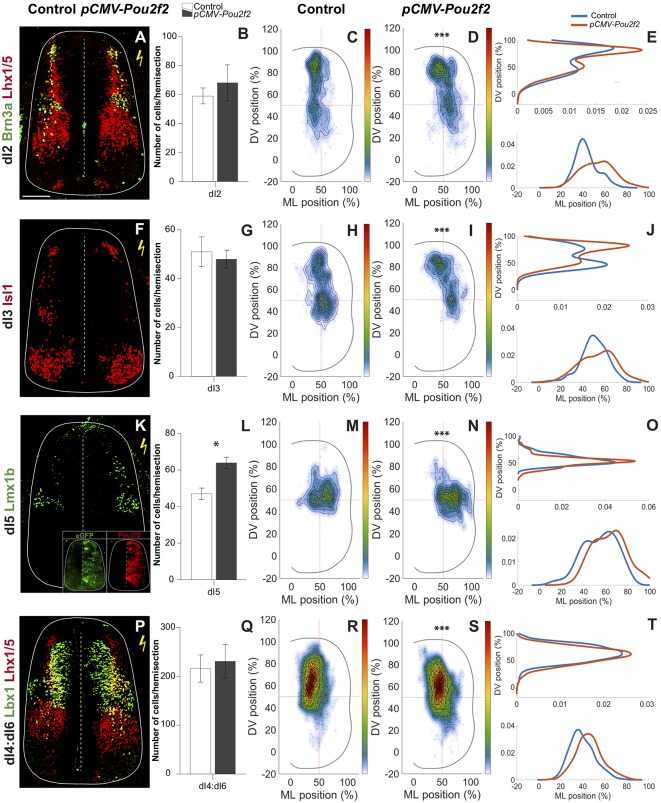
Dorsal interneuron distribution is altered after *Pou2f2* overexpression. Overexpression of *Pou2f2* in chicken embryonic spinal cord after electroporation at HH14–16 and immunolabelings 72 h after electroporation. Insets in **(K)** show immunodetection of GFP as electroporation control and of Pou2f2 as overexpression control. **(A,B)** Immunodetection and quantification of Brn3a^+^ (green) Lhx1/5^+^ (red) dI2 neurons on transverse sections of electroporated spinal cord at HH27–28. *Pou2f2* overexpression does not impact the number of dI2 interneurons. **(C–E)** Distribution analysis of dI2 interneurons in control or electroporated side of the chicken spinal cord. **(C,D)** Two-dimension distribution graphs (left) show integration of cell distribution from multiple sections of multiple embryos of each genotype. **(E)** One-dimension graphs compare density distribution in control (blue) and in electroporated spinal cord (red) on the dorso-ventral (DV; upper) or the medio-lateral (lower) axis of the spinal cord (see “Materials and Methods” section for details). Overexpression of *Pou2f2* alters the distribution of dI2 cells. **(C)** In control spinal cord, dI2 interneurons are distributed in a dorso-medial to ventro-medial fashion, with densely packed neurons close to the dI2 progenitor domain. **(D,E)** In electroporated spinal cord, dI2 cells migrate in a dorso-medial to ventro-lateral direction with more cells retained dorsally or located in a more lateral position (*p* ≤ 0.001). **(F,G)** Immunodetection and quantification of Isl1^+^ (red) dI3 neurons on transverse sections of electroporated spinal cord at HH27–28. All the Isl1^+^ cells dorsal to the motor columns are considered as dI3. The number of dI3 interneurons is not modified after *Pou2f2* overexpression. **(H)** In control spinal cord, dI3 are distributed in two closely connected clusters in a dorso-medial to ventro-medial direction. **(I,J)** In electroporated spinal cord, the dI3 interneurons are more dorsal and their lateral migration is increased (*p* ≤ 0.001). **(K,L)** Immunodetection and quantification of Lmx1b^+^ (green) dI5 neurons on transverse sections of electroporated spinal cord at HH27–28. The number of dI5 interneurons is significantly increased after *Pou2f2* overexpression. Moreover, their distribution is altered. **(M)** In control spinal cord, dI5 are distributed in two connected minor and major clusters both located in the medial tier of the spinal cord.** (N,O)** In electroporated spinal cord, the dI5 are located more laterally and slightly more ventrally (*p* ≤ 0.001). **(P,Q)** Immunodetection and quantification of Lbx1^+^ (green) Lhx1/5^+^ (red) dI4:dI6 neurons on transverse sections of electroporated spinal cord at HH27–28. *Pou2f2* overexpression does not alter the number of dI4 + dI6 neurons. **(R)** In control spinal cord, dI4 + dI6 are distributed in a dorso-medial to ventro-medial pattern.** (S,T)** In electroporated spinal cord, the dI4 + dI6 neurons organize in a dorso-medial to ventro-lateral fashion with more cells in a lateral position. Mean values ± SEM, *n* = 3, five sections per embryo (*n* > 710 cells per condition). *** = *p* ≤ 0.001. Scale bar = 100 μm.

Our data indicate that *Pou2f2* expression is increased in *OC*-deficient dIN populations. To assess whether Pou2f2 may contribute to the phenotype observed in *Hnf6/Oc2*^−/−^ embryos (Kabayiza et al., 2017), we mimicked this increase by overexpressing *Pou2f2* in chicken embryonic spinal cord. *Pou2f2* overexpression did not change the number of dI2 INs, co-labeled for Brn3a and Lhx1/5 (Figures 5A,B). In contrast, it significantly altered their distribution (Figures 5C–E). In HH27–28 control side of the spinal cord (Figure 5C), dI2 cells were distributed in a dorso-medial to ventro-medial manner, with densely packed cells in register with the dI2 progenitor domain. In contrast, in the electroporated side, the dI2 distributed in a dorso-medial to ventro-lateral direction with more cells retained dorsally in the densely packed cluster or located in a more lateral position (Figures 5C–E). As for dI2 INs, overexpression of *Pou2f2* in chicken embryonic spinal cord had no effect on the number of dI3 cells (Figures 5F,G). In the control side, dI3 INs distributed in two closely connected clusters in a general dorso-medial to ventro-medial direction (Figure 5H). However, in the electroporated side, a majority of dI3 INs remained more dorsal and the population extended laterally (Figures 5H–J). These observations indicate that increased Pou2f2 did not change dI2 or dI3 production but altered the migration of these populations in the dorsal spinal cord. In contrast, overexpression of *Pou2f2* in chicken embryonic spinal cord resulted in a significant increase in dI5 Lmx1b^+^ INs (Figures 5K,L), suggesting that Pou2f2 may promote dI5 production. Regarding their distribution, dI5 Lmx1b^+^ cells were located in the medial region along the DV axis of the spinal cord forming a minor medial cluster connected to a major lateral cluster on the medio-lateral axis in the control side of spinal cord (Figures 5M,O). However, this population was more lateral and slightly more ventral in electroporated spinal cord (Figures 5M–O). Finally, given the lack of a specific marker for dI4 and dI6 INs at this early developmental stage, the consequences of increased *Pou2f2* expression in the chick spinal cord were studied using Lbx1 and Lhx1/5 as dI4 and dI6 co-markers. As for dI2 and dI3 INs, Pou2f2 increase did not alter the number of dI4:dI6 cells (Figures 5P,Q). In control spinal cords, these cells were located in a dorso-medial to ventro-medial pattern. In contrast, in electroporated spinal cords, these INs were distributed in a dorso-medial to ventro-lateral direction with more cells in a lateral position (Figures 5R–T). Taken together, these data suggest that Pou2f2 is able to modulate the differentiation of dI5 and the distribution of dI2 to dI6 INs in the developing spinal cord. In particular, increased Pou2f2 resulted in a more dorsal and more lateral distribution of dIN populations.

### Pou2f2 Is Not Necessary for Proper Production of dINs

**Figure 6:**
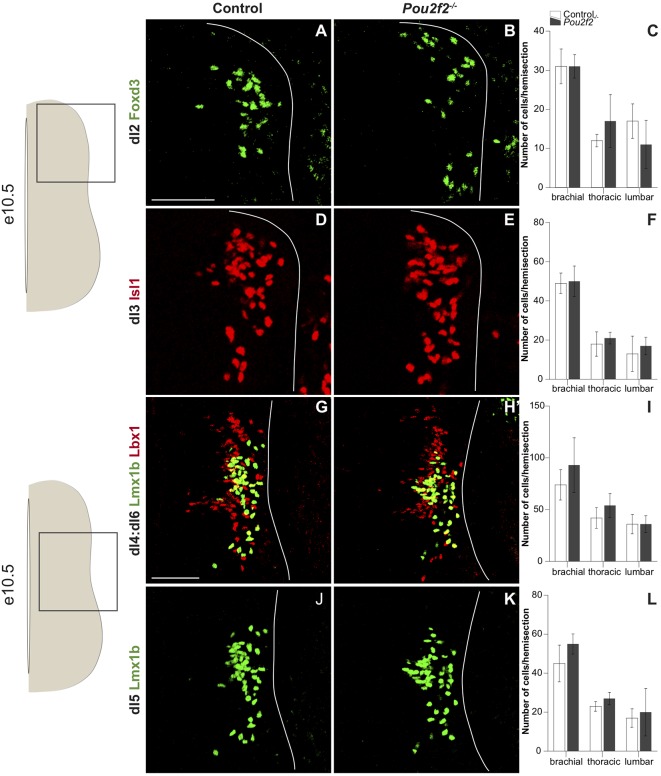
Dorsal interneuron production is normal at e10.5 in *Pou2f2* mutant embryos. **(A–L)** Immunodetection and quantification of Foxd3^+^ (green) dI2 neurons **(A–C)**, Isl1^+^ (red) dI3 neurons **(D–F)**, Lmx1b^+^ (green) Lbx1^+^ (red) dI4:dI6 neurons **(G–I)** and Lmx1b^+^ (green) dI5 neurons **(J–L)** on transverse sections of thoracic spinal cord at e10.5. Quantifications show comparable numbers of dI2 **(C)**, dI3 **(F)**, dI4:dI6 **(I)** and dI5 **(L)** cells in control or Pou2f2^−/−^ mutant embryos, indicating that the production of these populations is not affected in the absence of Pou2f2. The pictures show part of right hemisections as indicated on the schemes to the left. Solid lines delineate the spinal cord. Mean values ± SEM, *n* = 3. Scale bar = 100 μm.

Our overexpression data suggest that Pou2f2 is sufficient to modulate dI5 differentiation and dIN distribution. To determine whether Pou2f2 is necessary for these developmental processes, we assessed the differentiation and the distribution of each population of dI2-dI6 INs in *Pou2f2* mutant embryos (Corcoran et al., 1993). First, we determined whether early IN production was normal at e10.5 in the absence of Pou2f2. The number of dI2 cells, labeled for Foxd3, was similar in control and in *Pou2f2* mutants at each level of the spinal cord, as confirmed by cell quantification (Figures 6A–C), although the early distribution of these cells seemed different (Figures 6A,B). Similar analyses were carried out for dI3–dI6 populations. All those populations were produced in normal numbers in *Pou2f*^−/−^ embryos compared to control littermates (Figures 6D–L). Taken together, these results suggest that Pou2f2 is not necessary for the early steps of dIN differentiation.

### Pou2f2 Controls the Distribution of dI2 INs

**Figure 7:**
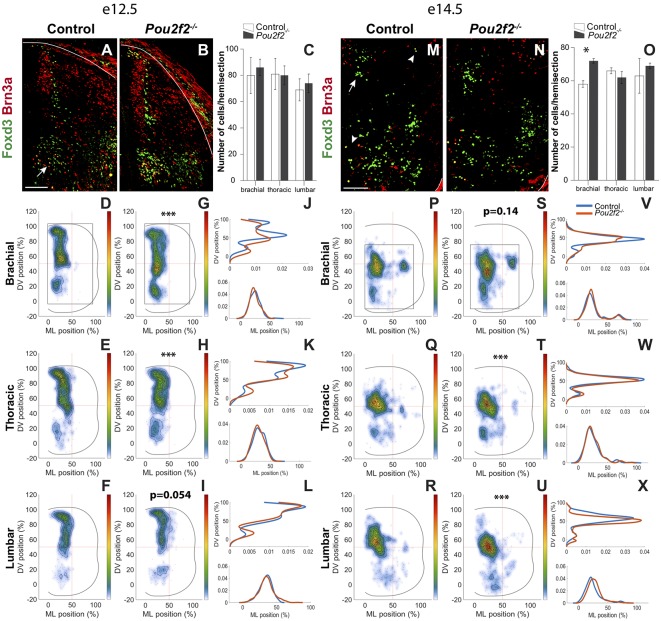
Pou2f2 regulates the distribution of dI2 interneurons.** (A–C)** Immunodetection and quantification of Foxd3^+^ (green) Brn3a^+^ (red) dI2 interneurons in control or *Pou2f2*^−/−^ mutant embryos at e12.5. The production of the Foxd3^+^ Brn3a^+^ dI2 interneurons is not altered in the absence of Pou2f2. **(D–L)** Distribution of dI2 interneurons on the transverse plane of the spinal cord in control or *Pou2f2*^−/−^ mutant embryos at e12.5. Two-dimension distribution graphs (left) show integration of cell distribution from multiple sections of multiple embryos of each genotype. One-dimension graphs (right) show density distribution in control (blue) or in *Pou2f2*^−/−^ embryos (red) on the DV (upper) or the medio-lateral (lower) axis of the spinal cord. The distribution of dI2 interneurons is affected in *Pou2f2*^−/−^ mutants, as dI2 cells migrate more ventrally, connecting the dorsal and ventral clusters at brachial level (*p* ≤ 0.001) and increasing the number of cells in the ventral cluster at thoracic (*p* ≤ 0.001) but not at lumbar levels (*p* = 0.0536). **(M–O)** Immunodetection and quantification of Foxd3^+^ (green) Brn3a^+^ (red) dI2 interneurons in control or *Pou2f2*^−/−^ mutant embryos at e14.5. The number of Foxd3^+^ Brn3a^+^ dI2 cells is increased at the brachial level, but not at more dorsal levels, in the absence of Pou2f2 (*p* ≤ 0.05). **(P–X)** Distribution of dI2 interneurons on the transverse plane of the spinal cord in control or Pou2f2^−/−^ mutant embryos at e14.5. In the absence of Pou2f2, dI2 neurons settle more ventrally and small lateral clusters are depleted at thoracic and lumbar levels (*p* ≤ 0.001). The pictures show part of right hemisections as indicated on the schemes in **(D,G)** and in **(P,S)**, respectively. Solid lines delineate the spinal cord. Mean values ± SEM, *n* = 3, five sections per level for each embryo (*n* > 2,803 cells per condition). * = *p* ≤ 0.05; *** = *p* ≤ 0.001. Scale bar = 100 μm.

However, the early migration of some dIN populations seemed affected at e10.5 (Figure 6). Given that OC factors regulate the distribution of dINs in the embryonic spinal cord (Kabayiza et al., 2017), that OC proteins repress *Pou2f2* expression in spinal INs (Figures 1–4 and Harris et al., 2019) and that Pou2f2 is able to modulate the position of these cells (Figure 5), we assessed whether the loss of *Pou2f2* impacts on dIN distribution at e12.5 and e14.5, i.e., in the course of interneuron migration and when migration of these cells in the transverse plan of the spinal cord is completed, respectively. To discriminate dI2 INs located in ventral regions from V1 cells, which also produce Foxd3, dI2 were additionally labeled for Brn3a. As observed at e10.5, the number of dI2 INs was not altered at e12.5 in the absence of Pou2f2 (Figures 7A–C). In control embryos, a majority of dI2 cells distributed in a medial stream of cells migrating from the dI2 progenitor domain towards the ventral region of the spinal cord and covering 60% of the ventro-dorsal axis. A second cluster of dI2 neurons expressing high amounts of Brn3a (arrow in Figure 7A) was located ventrally in the vicinity of the Foxd3^+^ V1 INs (Figures 7D–F). In *Pou2f2*^−/−^ mutant embryos, cells producing Foxd3 and Brn3a were detected in similar regions (Figure 7B). However, distribution analysis showed that dI2 INs migrate more ventrally in the absence of *Pou2f2*, connecting the dorsal and ventral clusters at brachial level and increasing the number of cells in the ventral cluster at thoracic and lumbar levels (Figures 7G–L).

At e14.5, the number of dI2 cells at brachial level was mildly but significantly increased in *Pou2f2*^−/−^ mutant embryos (Figures 7M–O). In control embryos, a main dI2 cluster settled in the intermediate part of the medial spinal cord (arrow in Figures 7M,P–R) whereas two smaller subsets located in a more ventral or lateral position, respectively (arrowheads in Figures 7M,P–R). In mutant embryos, the cells distributed in a similar pattern to control littermates although, reminiscent of e12.5, dI2 were located more ventrally and the small lateral cluster was depleted at thoracic and lumbar levels (Figures 7N,S–X). Taken together, these observations suggest that Pou2f2 regulates the distribution of dI2 INs.

### Pou2f2 Controls the Distribution of dI3 INs

**Figure 8:**
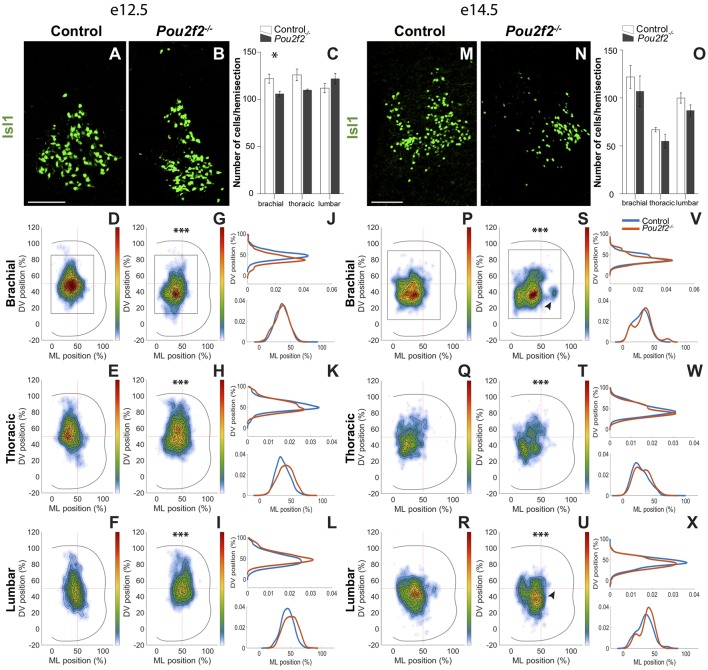
Pou2f2 regulates the distribution of dI3 interneurons.** (A–C)** Immunodetection and quantification of Isl1^+^ (green) dI3 interneurons in control or *Pou2f2*^−/−^ mutant embryos at e12.5. The production of the Isl1^+^ dI3 interneurons is not altered in the absence of Pou2f2 except for a slight decrease at brachial level (*p* ≤ 0.05). **(D–L)** Distribution of dI3 interneurons on the transverse plane of the spinal cord in control or Pou2f2^−/−^ mutant embryos. Two-dimension distribution graphs (left) show integration of cell distribution from multiple sections of multiple embryos of each genotype. One-dimension graphs (right) show density distribution in control (blue) or in *Pou2f2*^−/−^ embryos (red) on the DV (upper) or the medio-lateral (lower) axis of the spinal cord. In control spinal cord, dI3 are distributed as a single homogeneous cluster. In *Pou2f2*^−/−^ mutant embryos, dI3 cells are relatively more ventral at brachial level or lateral at thoracic and lumbar levels (*p* ≤ 0.001). **(M–O)** Immunodetection and quantification of Isl1^+^ (green) dI3 interneurons in control or *Pou2f2*^−/−^ mutant embryos at e14.5. The number of Isl1^+^ dI3 cells is unaffected in the absence of Pou2f2. **(P–X)** Distribution of dI3 interneurons on the transverse plane of the spinal cord in control or *Pou2f2*^−/−^ mutant embryos at e14.5. Still organized in a single cluster, dI3 in control embryos settle in the intermediate to ventral regions of the spinal cord. More ventral and lateral distribution is detected in Pou2f2 depleted embryos (*p* ≤ 0.001). A tiny ectopic cluster is present at brachial level or absent at lumbar level. The pictures show part of right hemisections as indicated on the schemes in **(D,G)** and in **(P,S)**, respectively. Mean values ± SEM, *n* = 3, five sections per level for each embryo (*n* > 3,724 cells per condition). * = *p* ≤ 0.05; *** = *p* ≤ 0.001. Scale bar = 100 μm.

Except for a mild but significant reduction at e12.5 at brachial level, the number of dI3 INs characterized by the expression of Isl1 was not affected in the absence of *Pou2f2* (Figures 8A–C,M–O). At e12.5, the dI3 cells gathered as a single homogeneous cluster in the intermediate region of the spinal cord, resulting in a Gaussian-like distribution along the DV and the medio-lateral axes (Figures 8A,D–F). In *Pou2f2*^−/−^ spinal cords, the population was slightly more ventral at brachial level and more lateral at thoracic and lumbar levels, respectively, with a more diffuse distribution in the center of the cluster (Figures 8B,G–L). Two days later, still gathered in a single cluster, dI3 neurons settled in the intermediate region of the spinal cord. The dI3 INs located more ventral and lateral in the absence of *Pou2f2* (Figures 8P–X). In addition, a tiny ectopic dI3 cluster was detected at brachial level, whereas a similarly located cluster was missing at lumbar level (arrowheads in Figures 8S,U). These observations suggest, as observed for dI2 cells, that Pou2f2 controls the distribution of dI3 INs in the developing spinal cord.

### Pou2f2 Controls the Differentiation and the Distribution of dI5 INs

**Figure 9:**
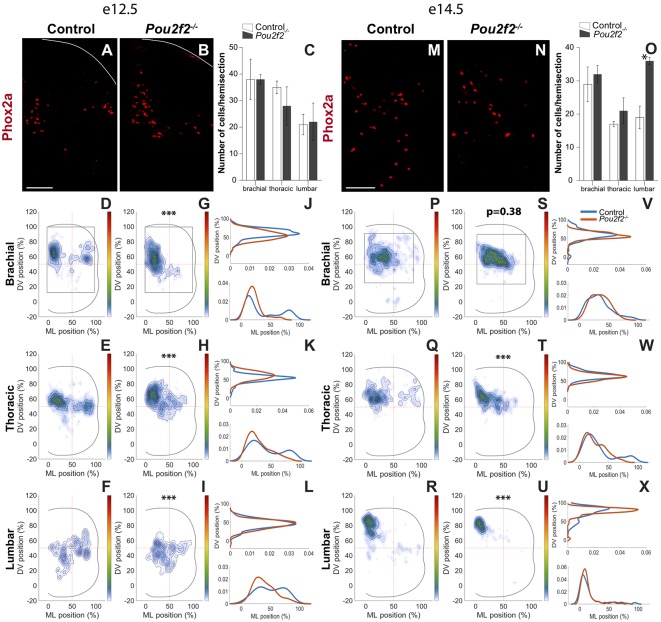
Pou2f2 regulates the distribution of dI5 Phox2a subset.** (A–C)** Immunodetection and quantification of dI5 Phox2a^+^ (red) subset in control or *Pou2f2*^−/−^ mutant embryos at e12.5. The production of the Phox2a^+^ dI5 interneurons is not altered in the absence of Pou2f2. **(D–L)** Distribution of dI5 Phox2a^+^ cells on the transverse plane of the spinal cord in control or *Pou2f2*^−/−^ mutant embryos. Two-dimension distribution graphs (left) show integration of cell distribution from multiple sections of multiple embryos of each genotype. One-dimension graphs (right) show density distribution in control (blue) or in *Pou2f2*^−/−^ embryos (red) on the DV (upper) or the medio-lateral (lower) axis of the spinal cord. The distribution of dI5 Phox2a^+^ subset is dramatically affected in *Pou2f2*^−/−^ mutants. In control embryos, dI5 Phox2a^+^ cells are organized in a major medial and a minor lateral cluster that progressively coalesce in more caudal regions. In Pou2f2-deficient spinal cords, a unique cluster located in a medial position is observed (*p* ≤ 0.001). **(M–O)** Immunodetection and quantification of dI5 Phox2a^+^ (red) subset in control or *Pou2f2*^−/−^ mutant embryos at e14.5. The number of Phox2a^+^ dI5 cells is significantly increased at lumbar level (*p* ≤ 0.05). **(P–X)** Distribution of dI5 Phox2a^+^ neurons on the transverse plane of the spinal cord in control or *Pou2f2*^−/−^ mutant embryos at e14.5. In control embryos, dI5 Phox2a^+^ gathered in a main medial cluster whereas, in *Pou2f2*^−/−^ spinal cords, cells were organized in a more compact cluster shifted laterally at brachial and thoracic levels and more dorsally at lumbar level (*p* ≤ 0.001). The pictures show part of right hemisections as indicated on the schemes in **(D,G)** and in **(P,S)**, respectively. Solid lines delineate the spinal cord. Mean values ± SEM, *n* = 3, five sections per level for each embryo (*n* > 930 cells per condition). * = *p* ≤ 0.05; *** = *p* ≤ 0.001. Scale bar = 100 μm.

In the mouse developing spinal cord, from e11.0 onwards, Lmx1b is present in the dI5 and in the late born dIL^B^ INs. As early- and late-born Lmx1b^+^ cells are difficult to discriminate for reproducible distribution studies, we restricted our analyses to the Phox2a^+^ dI5 subset at e12.5 and e14.5 (Figure 9). Absence of *Pou2f2* did not impact the number of dI5 Phox2a^+^ INs except for the lumbar level at e14.5, which showed a significant increase (Figures 9A–C,M–O). In control embryos at e12.5, Phox2a^+^ dI5 distributed in 2 main clusters on the medio-lateral axis of the spinal cord, with a major medial cluster and a minor lateral cluster that trended to coalesce in more caudal sections (Figures 9A,D–F). In *Pou2f2*^−/−^ embryos, dI5 distribution was dramatically changed with a majority of cells organized in a unique cluster located in a medial position (Figures 9B,G–L). At e14.5, control Phox2a^+^ dI5 gathered in a main medial cluster located more dorsally in more caudal sections (Figures 9M,P–R). In mutant embryos, cells were more compact but occupied a more lateral position at brachial and thoracic levels, and a more dorsal position at lumber level (Figures 9N,S–X). These observations indicate that Pou2f2 regulates the differentiation and the distribution of the Phox2a^+^ dI5 subpopulation.

### Pou2f2 Controls the Distribution of dI6 INs

**Figure 10:**
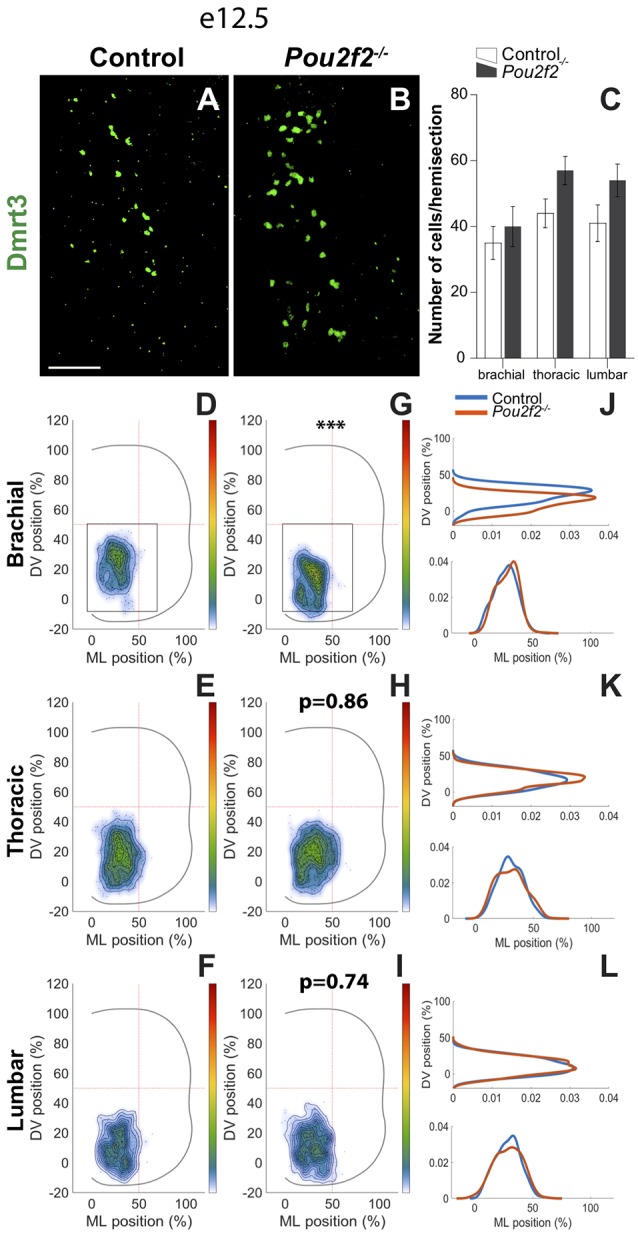
Pou2f2 regulates the distribution of the dI6 Dmrt3 subset.** (A–C)** Immunodetection and quantification of dI6 interneuron Dmrt3^+^ (green) subset in control or *Pou2f2*^−/−^ mutant embryos at e12.5. The production of the Dmrt3^+^ dI6 interneurons is not altered in the absence of Pou2f2. **(D–L)** Distribution of dI6 Dmrt3^+^ cells on the transverse plane of the spinal cord in control or *Pou2f2*^−/−^ mutant embryos. Two-dimension distribution graphs (left) show integration of cell distribution from multiple sections of multiple embryos of each genotype. One-dimension graphs (right) show density distribution in control (blue) or in *Pou2f2*^−/−^ embryos (red) on the DV (upper) or the medio-lateral (lower) axis of the spinal cord. Dmrt3^+^ dI6 distribution is affected in *Pou2f2*^−/−^ mutants at brachial level as these cells settle more ventrally relatively to control littermates (*p* ≤ 0.001). The pictures show part of right hemisections as indicated on the schemes in **(D,G)**. Mean values ± SEM, *n* = 3, five sections per level for each embryo (*n* > 1,700 cells per condition). *** = *p* ≤ 0.001. Scale bar = 100 μm.

**Figure 11:**
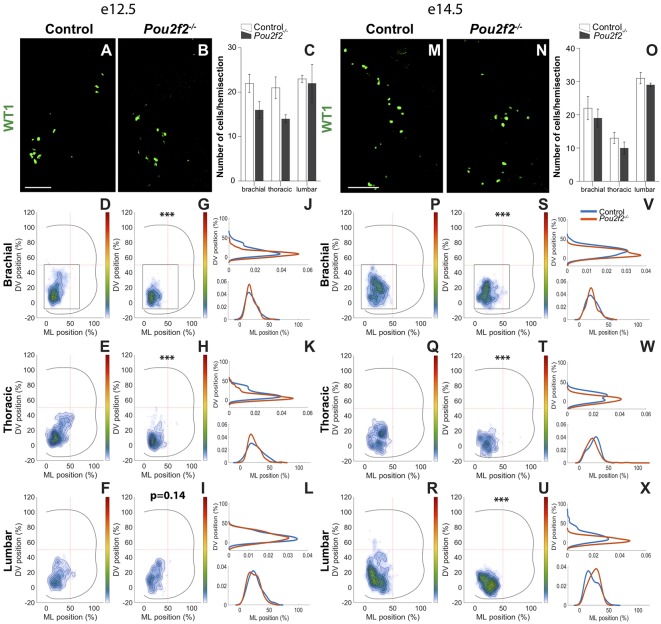
Pou2f2 regulates the distribution of dI6 Wt1 subset.** (A–C)** Immunodetection and quantification of dI6 Wt1^+^ (green) subset in control or *Pou2f2*^−/−^ mutant embryos at e12.5. The production of the Wt1^+^ dI6 interneurons is not altered in the absence of Pou2f2. **(D–L)** Distribution of dI6 Wt1^+^ neurons on the transverse plane of the spinal cord in control or *Pou2f2*^−/−^ mutant embryos. Two-dimension distribution graphs (left) show integration of cell distribution from multiple sections of multiple embryos of each genotype. One-dimension graphs (right) show density distribution in control (blue) or in *Pou2f2*^−/−^ embryos (red) on the dorso-ventral (upper) or the medio-lateral (lower) axis of the spinal cord. In control spinal cord, the dI6 Wt1^+^ subset is localized in the ventro-medial tier. In *Pou2f2*^−/−^ mutants, at brachial and thoracic levels, cells were more densely clustered and located more ventrally and medially (*p* ≤ 0.001). **(M–O)** Immunodetection and quantification of dI6 Wt1^+^ (green) subset in control or *Pou2f2*^−/−^ mutant embryos at e14.5. The size of the Wt1^+^ dI6 subset is not altered in the absence of Pou2f2. **(P–X)** Distribution of dI6 Wt1^+^ cells on the transverse plane of the spinal cord in control or *Pou2f2*^−/−^ mutant embryos at e14.5. Reminiscent of e12.5, dI6 Wt1^+^ subset remained more ventrally and more densely packed in the absence of Pou2f2 (*p* ≤ 0.001). The pictures show part of right hemisections as indicated on the schemes in **(D,G)** and in **(P,S)**, respectively. Mean values ± SEM, *n* = 3, five sections per level for each embryo (*n* > 635 cells per condition). *** = *p* ≤ 0.001. Scale bar = 100 μm.

At e12.5 and e14.5, two partially overlapping dI6 subpopulations are characterized by the presence of Dmrt3 or WT1, respectively. Absence of *Pou2f2* had no impact on dI6 cell number of each subpopulation but resulted in alterations in Dmrt3^+^ (Figures 10A–C) and in WT1^+^ (Figures 11A–C) dI6 distribution. At e12.5, control Dmrt3^+^ dI6 INs were located in the ventro-medial part of the spinal cord (Figures 10A,D–F). In *Pou2f2*^−/−^ embryos, Dmrt3^+^ INs settled more ventrally at brachial level than in mutant embryos (Figures 10B,G–L). Due to technical restrictions, we limited our distribution analysis of Dmrt3^+^ dI6 subset to e12.5.

In control embryos at e12.5, the WT1^+^ dI6 subset was located, similarly to the Dmrt3^+^ subpopulation, in the ventro-medial part of the spinal cord (Figures 11A,D–F). In *Pou2f2*^−/−^ embryos, WT1^+^ dI6 INs were more densely packed and were located more ventrally than in control littermates (Figures 11B,G–L). Consistently, at e14.5, WT1^+^ cells remained more densely packed and more ventral than in control littermates (Figures 11M–X). These observations indicate that Pou2f2 controls some aspects of dI6 distribution in the developing spinal cord.

## Discussion

During spinal cord development, proper cell migration is critically required for adequate integration of post-mitotic neurons into specific neural circuits. Recent studies demonstrated the importance of correct population and subpopulation distribution for spinal circuitry formation (Sürmeli et al., 2011; Tripodi et al., 2011; Goetz et al., 2015; Hinckley et al., 2015; Lu et al., 2015; Bikoff et al., 2016; Hilde et al., 2016). Although the involvement of an extensive amount of guidance molecules in the regulation of neuronal migration has been well characterized (Chen, 2019), the genetic programs that control the production of these guidance molecules and the selective responsiveness of distinct neuronal populations to these cues are still poorly understood, particularly in the spinal cord. Here we provide evidence that a genetic cascade composed of OC factors and their downstream target *Pou2f2* controls the distribution of dINs in the developing spinal cord.

### OC Factors Repress *Pou2f2* Expression in the Developing Spinal Cord

The diversification and the distribution of dIN have been recently shown to be regulated by OC transcription factors (Kabayiza et al., 2017). In an effort to identify downstream effectors involved in this process, we uncovered genes regulated by OC factors in the embryonic spinal cord. Only a limited number of genes coding for guidance cues were identified. Moreover, none of the candidate genes known to control neuronal migration were confirmed to be under OC protein regulation in the spinal cord. The outcome of this analysis suggests that the migration of each spinal neuronal population may be regulated by diverse micro-environmental cues and receptors, instead of a generic mechanism common to all populations. The diversity of migration routes and endpoints (Lu et al., 2015; Lai et al., 2016; Chen, 2019) is consistent with this hypothesis. However, expression of the *Pou2f2* transcription factor appeared to be repressed by the OC factors in the embryonic spinal cord. *OC* inactivation did not result in a significant increase in the number of dINs containing Pou2f2, unless for the dI6 Dmrt3^+^ subset. In contrast, cells expressing *Pou2f2* in control embryos show increased *Pou2f2* levels in *OC* mutant spinal cord. Thus, OC factors seem to moderate *Pou2f2* expression in its endogenous domain rather than preventing an ectopic activation in other dINs subpopulations. However, OC factors are supposed to mainly behave as transcriptional activators (Jacquemin et al., 2000, 2003; Pierreux et al., 2004; Roy et al., 2012). Therefore, our observations suggest an indirect regulation of *Pou2f2* expression by the OC factors. Alternatively, we can not exclude that OC factors may exert a dual role as both a transcriptional activator or a repressor depending on the cell type, as previously shown for Gli3, which can act both as an activator or as a repressor during different phases of zebrafish CNS patterning (Tyurina et al., 2005), or Arx known for its bifunctional activity during Xenopus forebrain development (Seufert et al., 2005). Identification of the 5′ sequences of the embryonic spinal *Pou2f2* transcripts (Harris et al., 2019) and of the regulating sequence controlling the expression of *Pou2f2* in the developing spinal cord would be required to unveil the mechanisms whereby OC regulate *Pou2f2* expression. Nevertheless, our analysis uncovered *Pou2f2* as a downstream target of OC factors in the dINs.

### An OC-Pou2f2 Genetic Cascade Regulates the Distribution of dINs

To assess whether Pou2f2 may regulate dIN distribution, we increased *Pou2f2* expression in the spinal cord using chicken embryonic electroporation. *Pou2f2* overexpression resulted in dINs mislocalization without any change in their cell number, except for the dI5 Phox2a subset. These alterations are reminiscent of dIN alterations observed in the absence of OC factors (Kabayiza et al., 2017), even if *in ovo* electroporation constrains the analysis to developmental stages earlier than those studied in the mouse, which hinders the evaluation of terminal settling position of the dINs. In *Hnf6/Oc2*^−/−^ embryos, dI3 INs displayed altered migration trajectory (Kabayiza et al., 2017). Similarly, after *Pou2f2* overexpression, chicken dI3 migration pathway was altered. Furthermore, the dI5 INs were mislocalized after *Pou2f2* overexpression, as observed for the Phox2a-positive dI5 subset in *Hnf6/Oc2*^−/−^ embryos. However, these changes in distribution were not strictly identical, likely owing to differences in the timing, the spatial extent and the level of *Pou2f2* overexpression between the mouse and the chicken models.

Consistently, *Pou2f2* inactivation affected dIN distribution with only mild effects on the size of each population. Different types of localization defects were observed in *Pou2f2*^−/−^ embryos. First, the ventral part of dI2 INs, dI6 Dmrt3 and dI6 Wt1 INs subsets settled in more ventral localizations. These cells seemed to be more advanced in their migration process, reaching faster and even going beyond their targeted localization. Second, dI5 Phox2a subset showed the most dramatic distribution defect, as its organization was altered from two clusters to a unique main cluster. Variations in these localization defects suggest, as proposed above, that different migration cues are affected in each dIN population. Nevertheless, these observations demonstrate that Pou2f2 contributes to regulating dIN migration. Furthermore, a comparison of distribution alterations between *Pou2f2* and *OC* mutant embryos points to a possible contribution of Pou2f2 downstream of OC factors in the control of dIN migration. Indeed, *Pou2f2* depletion resulted in a relatively loose dI3 population, whereas dI3 were more compactly distributed in *OC* mutants. Inversely, Phox2a-positive dI5 were more densely packed along the DV axis in *Pou2f2* mutants but more spread in *OC* mutants. However, although depletion or overexpression of *Pou2f2* might have opposite effects on the expression of its downstream guidance cue targets, these will not necessarily lead to opposite effects on cellular migration. Nevertheless, we propose that a genetic cascade involving OC and Pou2f2 transcription factors controls the distribution of dI2-dI6 INs in the developing spinal cord.

### Pou2f2 Acts as a Regulator of dIN Distribution

Although Pou2f2 has been previously described as a transcription factor regulating the maturation of B cell precursors into immunoglobulin-secreting B cells (Corcoran et al., 1993), it is also expressed in the central nervous system during development and in the adult brain (Hatzopoulos et al., 1990; Lillycrop and Latchman, 1992; Stoykova et al., 1992; Camós et al., 2014). A high-resolution *in situ* hybridization analysis detected *Pou2f2* expression at e13.5 in the midbrain (Thompson et al., 2014), a time point that corresponds to late neurogenesis (Bayer et al., 1995). Consistently, we observed the presence of Pou2f2 in differentiating dI2-dI6 INs of the developing spinal cord. In mouse ES cells undergoing neuronal differentiation, Pou2f2 could act as a bifunctional regulator of differentiation depending on the predominant isoform (Theodorou et al., 2009), although the isoforms considered are different from those identified in the developing spinal cord (Harris et al., 2019). *Pou2f2* inactivation is associated with neonatal lethality (Corcoran et al., 1993), suggesting its involvement in the development of vital nervous functions. However, its role in neuronal CNS development had not been explored yet. Our analysis of several dIN populations suggested that Pou2f2 contributes to dIN migration. Potential Pou2f2 targets in the CNS are currently unknown. Recent studies have uncovered the presence of Pou2f2 in several tumors including pancreatic and gastric cancers, the latter having a high frequency of invasiveness and metastasis. Interestingly, Pou2f2 has been identified in a Nf-kB/Pou2f2/Robo1/Slit2 signaling cascade that promotes metastasis in gastric cancer cells (Wang et al., 2016). In this cascade, Pou2f2 directly regulates the *Robo1* gene, a member of the ROBO family, and activates its expression. Several studies point out the importance of the Slit/Robo repulsive signals in neuronal migration (Wu et al., 1999; Causeret et al., 2002; Andrews et al., 2006; Di Meglio et al., 2008). In the developing forebrain, through semaphorin-neuropilin/plexin signaling modulation, Robo1 guides interneuron migration through the subpallium and into the cortex (Hernández-Miranda et al., 2011). Oppositely to Robo1 and Robo2 receptors, Robo3 does not bind Slits but instead interacts with Dcc and Netrin-1. This mechanism of action mediates attraction (Zelina et al., 2014) and, probably through Dcc and Robo3, Netrin-1 promotes dINs migration (Junge et al., 2016). Additionally, Slit/Robo repellent signaling, in parallel with Netrin-1/DCC attractive cues, ensures correct positioning of spinal motor neurons in the ventral horn (Kim et al., 2015). Hence, Pou2f2 may regulate *Robo1* expression in dIN populations and thereby promote the production of repulsive guidance cues. Interestingly, in *Pou2f2* mutants, several dIN populations progress deeper than expected in the ventral spinal cord. Secreted Slits are produced by the ventral spinal cord, which makes it a repulsive territory for neurons expressing Robo1 and Robo2 receptors (Long et al., 2004). In *Pou2f2*-depleted dINs, *Robo1* expression could be downregulated, resulting in altered responsiveness to repellent Slit signals that would authorize a more ventral localization.

Perturbations in neuronal position and/or migration during development result in heavy conditions such as “type I” lissencephaly related to the mislocalization of cortical neurons (Hirotsune et al., 1998; Vallee and Tsai, 2006) or constitute a risk factor for schizophrenia (Tomita et al., 2011). Interestingly, two other POU domain transcription factors, Pou3f2 and Pou3f3 are suspected to be involved in schizophrenia due to their effect on cortical neuron migration (Potkin et al., 2009; Dominguez et al., 2013). Even if diseases associated with neuronal positioning defects have not been described in the spinal cord yet, it has been recently demonstrated that interneuron localization is critical for proper spinal circuit formation. Indeed, the settlement position of inhibitory V1 IN subsets correlates with their input connectivity (Bikoff et al., 2016). Similarly, stereotypical motor neuron localization is of importance for sensory connection establishment. In fact, proprioceptors target specific DV tiers of the spinal cord without regard to the identity of motor neurons present (Sürmeli et al., 2011). Hence, the adequate position of motor neurons is initially of greater importance for sensory-motor connectivity than their identity. Alterations in dIN settling position also cause impairments of sensory-motor signaling, as demonstrated for Satb2^+^ lamina V inhibitory sensory relay neurons. Loss of Satb2 resulted in altered medio-lateral position of these dINs, which subsequently affected the proprioceptive innervation on those cells (Hilde et al., 2016). Soma localization may also influence the ingrowth of sensory afferents to the dorsal horn. Indeed, selective depletion of *Bcl11a* in dINs alters, besides its role in neuron morphogenesis, the traveling of sensory fibers to their spinal targets (John et al., 2012). Other transcription factors are known to influence dIN migration, including Lmx1b and its downstream targets *Drg11* and *Rnx*, the depletion of which leads to dI5 aberrant localization (Ding et al., 2004). Here we demonstrated that dINs show aberrant soma settling position when *Pou2f2* is either overexpressed or depleted. Interestingly, the dIN populations affected in the absence of Pou2f2 are involved either in the modulation of motor neuron activity (Andersson et al., 2012; Bui et al., 2013; Goetz et al., 2015; Satoh et al., 2016) or of ventral premotor interneuron activity (Levine et al., 2014; Hilde et al., 2016), or in the presynaptic inhibition of proprioceptive sensory neuron terminals that hinder sensory inputs onto motor neurons (Betley et al., 2009; Fink et al., 2014; Zhang et al., 2017). Whether alterations in *Pou2f2* mutant embryos affect the activity of the sensory-motor circuits of the spinal cord remain to be determined.

## Data Availability Statement

The datasets (microarray comparison of control and *Hnf6/Oc2*^−/−^ spinal cords at e11.5) for this study can be found in the GEO repository (accession number: GSE117871; https://www.ncbi.nlm.nih.gov/geo/query/acc.cgi?acc=GSE117871).

## Ethics Statement

The animal study was reviewed and approved by Animal Welfare Committee, UCLouvain.

## Author Contributions

GM, AH and FC designed the experiments. LC provided critical reagents and contributed to initial discussions. GM and AH performed the experiments. BJ contributed to data analyses and all the authors discussed the data. GM and FC drafted the manuscript.

## Conflict of Interest

The authors declare that the research was conducted in the absence of any commercial or financial relationships that could be construed as a potential conflict of interest.
